# Sleep quality among undergraduate medical students in Rwanda: a comparative study

**DOI:** 10.1038/s41598-023-27573-9

**Published:** 2023-01-06

**Authors:** Amon Nsengimana, Eric Mugabo, Japhet Niyonsenga, Jean Claude Hategekimana, Emmanuel Biracyaza, Renauvat Mutarambirwa, Emile Ngabo, Richard Nduwayezu

**Affiliations:** 1 School of Medicine and Pharmacy, College of Medicine and Health Sciences, University of Rwanda, Kigali, Rwanda; 2 School of Rehabilitation, Faculty of Medicine, University of Montreal, Montreal, QC Canada; 3 Oazis Health, Kigali, Rwanda

**Keywords:** Psychology, Health care, Neurology

## Abstract

Despite the abundance of literature highlighting poor sleep quality among medical students and its detrimental impact on their mental well-being and academic performance, no study has been conducted to investigate the sleep quality of undergraduate medical students in Rwanda to date. Therefore, this study sought to determine the magnitude of sleep quality of undergraduate medical students in Rwanda and to compare the scores of seven components of sleep quality across classes. This cross-sectional study was conducted among 290 undergraduate medical students aged 18–35 years (mean = 24, SD = 2.9) randomly recruited countrywide from 1st November 2021 to 1st March 2022. The questionnaire was self-administered with 2 sections: characteristics of medical students, and Pittsburgh Sleep Quality Index (PSQI). The Pearson Chi-square test was used to test whether the categories of seven components of sleep quality differ between classes, then ANOVA followed by the post hoc test was used to test if the seven components and global score of Pittsburgh Sleep Quality Index differ between classes. The results revealed that the global PSQI mean score was 7.73 (SD = 2.83), with fifth-year medical students reporting the highest PSQI mean score (M = 8.44, SD = 2.77), followed by first-year (M = 8.15, SD = 3.31). One-way ANOVA showed that the global PSQI score (F = 2.76, p = 0.028), subjective sleep quality (F = 3.35, p = 0.011), habitual sleep efficiency (F = 10.20, p < 0.001), and daytime dysfunction (F = 3.60, p = 0.007) were significantly different across classes. Notably, the post hoc test revealed significant scores differences in the global PSQI score between class II and V (p = 0.026), in subjective sleep quality between class I and II (p = 0.043), and between class I and IV (p = 0.016); habitual sleep efficiency between class V and all other classes (p < 0.001); and daytime dysfunction between class III and IV (p = 0.023). This paper concludes by arguing that poor sleep quality is highly prevalent among medical students in Rwanda, with final and first-year students reporting the poorest sleep quality. There were significant differences across classes in the global PSQI, subjective sleep quality, habitual sleep efficiency, and daytime dysfunction. Intervention approaches such as sleep education, behavioral changes, and relaxing techniques are recommended to address contributing factors and ultimately maximize the academic goals of Rwandan medical students.

## Introduction

Sleep quality is defined as one’s satisfaction with the sleep experience, which integrates aspects of sleep initiation, sleep maintenance, sleep quantity, and awakening refreshment^1^. Though the modern world ignores sleep, sleep is deemed a basic human need occupying a third of human life^2,3^. To humans, sleep is as important as breathing, eating, and urinating, and it is a necessary condition for physical and mental health^4^. Between 7 and 9 h of sleep is recommended for adults, and 9.5 h for teenagers^5,6^. The deviation from this range is regarded as a disruption of the sleep–wake cycle, which sometimes can lead to psychopathologies like sleeping disorders^7^. For medical students, good sleep quality is essential to have the optimum cognitive function, memory, and decision-making to excel and master their learning needs^8^. Unfortunately, several studies in the region and globally revealed that medical students are more vulnerable to poor sleep quality than non-medical students perhaps due to the long duration and high intensity of study^9–12^.

In Nepal, the prevalence of poor sleep quality among medical students was 44.2% which was higher compared to that of non-medical students, 30.3%^2^. More other global reviews reported that sleep disturbances affect 41% of participating students in Iran, 70% in Hong Kong, and 90% in China^2^. In Sub-Saharan African (SSA) countries like Ethiopia, and Nigeria, 62%, and 32.5% of medical students experienced poor sleep quality respectively^13,14^. Being over-stressed due to intensive learning periods with limited break time, having a daily schedule full of academic lectures, hospital activities, and painful emotional events such as dealing with patients who are severely suffering or dying are the most reported factors associated with poor sleep quality among medical students^13–15^. Furthermore. participation in internships and subclinical depression have been also reported to lead to sleep deprivation and thus poorly affect their sleep quality^9^. Though medical students may not consider sleep as a top priority due to their academic requirements, scholars have demonstrated that poor sleep quality is associated with psychiatric disorders, physical problems, impairment in job performance, decreased work efficiency and learning disability^16,17^. Further studies highlighted that sleep deprivation among medical students leads to sleepiness during the daytime, which may contribute to medical errors, road traffic accidents, and a decrease in academic performance^9,15,18^.

Medical students in post-conflict and low-income countries, such as Rwanda, may be more likely to report poor sleep quality associated with high mental health disorders and trans-generational trauma from their harmful past experiences or the experiences of their parents^19,20^, in addition to a wider range of circumstances holding a potential life threat to the population in this region, as seen in the burden of disease studies^21^. However, despite the substantial literature associating mental health problems such as depression, and post-traumatic stress disorders (PTSD) symptoms with poor sleep quality^9,22–24^, no study has assessed sleep quality among medical students in Rwanda ‘a post-genocide and low-income country’ where mental health disorders may be extremely high, yet this area of research is an important indicator of the quality of life and biopsychosocial well-being^14^. This study, therefore, aimed to determine the magnitude of sleep quality of undergraduate medical students in Rwanda and to compare the seven components and global score of Pittsburgh Sleep Quality Index between classes. In this study, we hypothesized that the medical students in year 1 would have worse sleep quality than those in the second year and above. Year 1 medical students may encounter a lot of challenges like new schedules, unfamiliar environments, and academic demands; therefore, cutting down their sleep to adapt to these heavy workloads^8^. This study will contribute to the scientific community and organizations working with medical students in Rwanda, “a post-conflict country” and similar settings by initiating effective intervention strategies. The findings of this study will help the concerned parties like policymakers or decision-makers in developing health strategies for promoting health through sleep hygiene programs not only among medical students but also among healthcare students in general.

## Methods

### Study design

A cross-sectional study design was conducted from 1st November 2021 to 1st March 2022 to assess the magnitude of sleep quality of undergraduate medical students in Rwanda and to compare the seven components and global score of Pittsburgh Sleep Quality Index between classes.

### Study population and settings

Rwanda is a Sub-Saharan African country located in the East Africa, covering a land area of 26,338 km^2^. The country is divided into four provinces (East, West, South, and North), as well as the capital city, Kigali. It is the country that has three Higher Learning Institutions including the University of Rwanda (UR); the Institute of Legal Practice and Development (ILPD), Rwanda’s dedicated postgraduate institution for legal education; and Rwanda Polytechnic (RP). Regarding medical programs, it has 3 medical schools including one for a public institution found at the University of Rwanda and 2 from private institutions: the University of Global Health Equity (UGHE) and the Adventist University of central Africa (AUCA). Rwandan medical school began at the former National University of Rwanda, which is now part of the University of Rwanda, College of Medicine and Health Sciences, School of Medicine, and Pharmacy. It is the largest medical school to date, offering both undergraduate and postgraduate programs. It has three campuses including the REMERA campus located in Kigali city, HUYE campus located in the southern province, and the GAKO campus located in the Eastern province.

### Sample size and sampling technique

A list of registered medical students for the academic year 2021–2022 was obtained from the Deans of the School of Medicine at each university. The total number of students was 1062, with 960 from UR, 66 from UGHE, and 36 from AUCA. The sample size was calculated using Yamane formula: ${\text{nY}}=\frac{{\text{N}}}{1+{{\text{Ne}}}^{2}}$, where “N” stands for ‘population size’, and “e” for Alpha level (e = 0.05) at the confidence interval of 95%. $nY=\frac{1062}{1+1062(0.0{5)}^{2}}=290$^25^. A disproportionate stratified sampling technique led to 253 participants enrolled from UR, 31 from UGHE, and 6 from AUCA.

### Data collection and measurements

Data were collected by trained data collectors from November 1st, 2021, to March 1st, 2022. All medical students enrolled at one of the three universities were included in the study. However, medical students under 18 years of age were not included as a tool used in this study to assess the sleep quality of medical students is designed for adults^22,26^. Moreover, as in previous studies, medical students with a chronic medical condition were excluded^27^. A chronic medical condition was a self-reported presence of one of the following: non-communicable disease or bronchial asthma^27^. Twelve of the 302 medical students approached were excluded because six had asthma, one had bipolar disorder, and five refused to participate. The study employed a self-administered questionnaire consisting of two sections: characteristics of medical students and the Pittsburgh Sleep Quality Index (PSQI) as described below:

### Characteristics of medical students

The characteristics of medical students were gender, age in years, university, class, scholarship, clinical rotations, accommodation, living with a roommate, smoking habits, class attendance, studying in team, and self-reported academic score.

### The Pittsburgh Sleep Quality Index (PSQI)

PSQI is a 19-item psychometric instrument used to assess participants’ sleep quality. This instrument presents a high internal consistency (α = 0.81) with a predictive validity cut-off point of a PSQI score > 5 showing 89.6% sensitivity and 86.5% specificity for identifying poor sleep quality^26^. It is an effective instrument that measures the quality and patterns of sleep-in adults, differentiating poor from good sleep quality by measuring different aspects of sleep disturbance during the past month^26^. In the context of the current study, this tool demonstrated a satisfactory internal consistency (Cronbach’s Alpha, α = 0.87). The PSQI has 19 items grouped into seven components: (a) subjective sleep quality, (b) sleep latency, (c) sleep duration, (d) habitual sleep efficiency, (e) sleep disturbances, (f) use of sleeping medications, and (g) sleep dysfunction^26^. Each component is scored on the 4-point Likert scale (0–3) with "0" implying no difficulty, whereas a "3" denotes extreme difficulty. These components together yield one global score, with a range of 0–21 points, "0" indicating no difficulty and "21" indicating severe difficulties in all areas. The scores of sleep disturbances, use of sleep medication, and daytime dysfunctions were classified as follows: 0 = none, 1 = mild, 2 = moderate, and 3 = severe to indicate the level of impairment in these components.

### Data analysis

Statistical Package for Social Sciences (SPSS) version 25.0 was used to conduct the statistical analysis. The results of the computation of descriptive statistics were shown as frequencies and percentages. The categories of each of the seven components of sleep quality were compared across all classes using the Pearson Chi-square test of independence (χ^2^), whilst the scores of the seven components of PSQI and global PSQI score were compared using ANOVA followed by Post Hoc Tukey Test. A two-tailed = 0.05 was employed in all analyses. In cases of multiple testing, we adjusted the α-level using a Bonferroni correction to avoid α-inflation^28^.

### Ethical declaration

The Helsinki Declaration of 1975, as amended in 2008, and the national and institutional committee on human experimentation's ethical requirements are both upheld by this study^29^. The ethical clearance was requested and approved by the Institutional Review Board of the College of Medicine and Health Sciences (IRB/CMHS) at the University of Rwanda under the reference number CMHS/IRB/284/2021.

### Consent to participate

Participants voluntarily consented to participate. The study participants provided their informed consent after adequate information about the study. Additionally, confidentiality was guaranteed.

## Results

### Socio-demographic characteristics of medical students

The study involved 290 medical students aged 18–35 with an average of 24, SD = 2.9. Male participants were 169 (58.3%) and 121 (41.7%) were female. A total of 253 (87.2%) were selected from UR, 31 (10.7%) from UGHE, and 6 (2.1%) from AUCA. More participants were in the 4th year, 120 (41.4%), while 58 (20%) were in the 5th year, 44 (15.2%) were in the 3rd year, 42 (14.5%) were in the 2nd year, and 26 (9%) were in the 1st year. The majority, 259 (89.3%) had a scholarship but 31 (10.7%) were self-sponsored. During the study period, 156 (53.8%) were in clinical rotations/clinical clerkship, 170 (58.6%) were accommodated on campus, slightly a quarter 67 (23.1%) rented houses while 53 (18.3%) were staying in their family. The majority, 202 (69.7%), were living with a roommate. The study found only 2 medical students (0.7%) who reported being smokers. The majority reported that they attend class regularly 264 (91%) while 26 (9%) were attending class irregularly. More than half participants, 154 (53.1%) reported that their academic grade was between 71 and 80%. When asked how many times they studied in a team, 210 (72.4%) participants reported sometimes, 36 (12.4%) all the time, 37 (12.8%) only when assigned to group work, whereas 7 (2.4%) reported having never studied in the team (Table 1).

**Table 1:** Characteristics of participants.

Variables	Frequency	Percent
**Gender**		
Male	169	58.3
Female	121	41.7
**Age (years)**		
≤ 18	20	6.9
19–24	205	70.7
25–30	58	20
31–35	7	2.4
**University**		
UR	253	87.24
UGHE	31	10.7
AUCA	6	2.1
**The academic level of study**		
1st year	26	9
2nd year	42	14.5
3rd year	44	15.2
4th year	120	41.4
5th year	58	20
**Scholarship**		
Yes	259	89.3
No	31	10.7
**Clinical rotation**		
Yes	156	53.8
No	134	46.2
**Accommodation**		
Campus	170	58.6
Rent house/room	67	23.1
Home	53	18.3
**Living partner**		
Living alone	25	8.6
Living with family	63	21.7
Living with roommate	202	69.7
**Smoking habits**		
No	288	99.3
Yes	2	0.7
**Class attendance**		
Regular attendance	264	91
Irregular attendance	26	9
**Studying in team**		
Sometimes	210	72.4
All the time	36	12.4
Only during group assignment	37	12.8
Never	7	2.4
**Self-reported academic score (%)**		
50–60	21	7.24
61–70	77	26.55
71–80	154	53.1
> 80	38	13.1

*UR*: University of Rwanda, *UGHE*: University of Global Health Equity, *AUCA*: Adventist University of Central Africa.

### Distribution of participants by sleep quality components and classes

Of the 290 students, 64 (22%) respondents reported having very good sleep quality whereas 167 (57.6%) experienced fairly good sleep quality. More than half, 155 (53.5%) reported falling asleep in more than 15 min, and, the majority, 202 (70%) slept 5–6 h per night. The habitual sleep efficiency was less than 65% among 197 (68%). Mild and moderate sleep disturbances were observed in 152 (52%) and 93 (32%) respectively. Only 19 (6.6%) used sleeping medications and 211 (73%) presented daytime sleep dysfunctions. Generally, the results indicated that 231 (80%) had poor sleep quality. Subjective sleep quality ranging from fairly good to very good among second-year students was standing at 27 (64.3%) and 10 (23.8%) respectively. However, 20 (34.5%) in the fifth year reported their subjective sleep quality as fairly bad. Second-year students were taking more minutes to fall asleep, there were 7 (25.4%) who took more than 30 min to fall asleep. Contrary, 106 (88.4%) students in the fourth year took 30 min or less. Most of the fifth-year medical students were sleeping 5–6 h per night, 44 (74.6%) while 6 (23.1%) in the first year were sleeping 6–7 h. The habitual sleep efficiency of 21 (50%) and 1 (2.4%) second-year students was ≥ 85% and 75–84% respectively. However, 53 (91.4%) in the fifth year was less than 65%. Sleep disturbances were more prevalent among fourth-year students, whereby a total of 73 (60.8%) and 33 (27.5%) had mild and moderate sleep disturbances respectively. The use of sleeping medications was more prevalent among the first-year students, 3 (11.5%) while sleep dysfunctions were more prevalent among third-year students 40 (91%). In general, poor sleep quality was more prevalent among first, and fifth-year medical students, 21 (80.8%) and 50 (86.2%) respectively. The Chi-square test revealed that subjective sleep quality, sleep duration, habitual sleep efficiency, sleep disturbances, and daytime dysfunction were significant across classes (χ^2^ = 34.17, p < 0.001; χ^2^ = 22.51; p = 0.032, (χ^2^ = 46.58, p < 0.001); (χ^2^ = 16.84, p < 0.032; χ^2^ = 33.35; p < 0.001) respectively (Table 2).

**Table 2:** Distribution of respondents by sleep quality components and classes.

Sleep quality component	Year I	Year II	Year III	Year IV	Year V	Pearson Chi-square	p-value
	n (%)	n (%)	n (%)	n (%)	n (%)	χ^2^	
**Subjective sleep quality**							
Very good	1 (3.8)	10 (23.8)	6 (13.6)	33 (27.5)	14 (24.1)	34.17	< 0.001***
Fairly good	16 (61.5)	27 (64.3)	31 (70.5)	69 (57.5)	24 (41.4)		
Fairly bad	7 (26.9)	5 (11.9)	4 (9.1)	15 (12.5)	20 (34.5)		
Very bad	2 (7.7)	0 (0)	3 (6.8)	3 (2.5)	0 (0)		
**Sleep latency (min)**							
≤ 15	12 (8.9)	20 (47.6)	16 (36.4)	59 (49.2)	28 (48.3)	10.47	0.575
16–30	12 (46.2)	15 (35.7)	22 (50)	47 (39.2)	23 (39.7)		
31–60	0 (0)	6 (14.3)	6 (13.6)	10 (8.3)	5 (8.6)		
> 60	2 (22.2)	1 (11.1)	0 (0)	4 (3.3)	2 (3.4)		
**Sleep duration (h)**							
> 7	0 (0)	2 (4.9)	0	8 (6.7)	0 (0)	22.51	0.032*
6–7	6 (23.1)	9 (22)	3 (6.8)	13 (10.8)	8 (13.5)		
5–6	13 (50)	27 (65.9)	33 (75)	85 (70.8)	44 (74.6)		
< 5	7 (26.9)	3 (7.3)	8 (18.2)	14 (11.7)	7 (11.9.)		
**Habitual sleep efficiency (%)**							
≥ 85	12 (46.2)	21 (50)	22 (50)	28 (23.3)	5 (8.6)	46.58	< 0.001***
75–84	1 (3.8)	1 (2.4)	0 (0)	0 (0)	0 (0)		
65–74	0 (0)	0 (0)	0 (0)	3 (2.5)	0 (0)		
< 65	13 (50)	20 (47.6)	22 (50)	89 (74.2)	53 (91.4)		
**Sleep disturbances**							
None	5 (19.2)	5 (11.9)	6 (13.6)	14 (11.7)	15 (25.9)	16.84	0.032*
Mild	9 (34.6)	27 (64.3)	21 (47.7)	73 (60.8)	22 (37.9)		
Moderate	12 (46.2)	10 (23.8)	17 (38.6)	33 (27.5)	21 (36.2)		
Severe	0 (0)	0 (0)	0 (0)	0 (0)	0 (0)		
**Sleeping medication**							
None	23 (88.5)	40 (95.2)	42 (95.5)	110 (91.7)	56 (96.6)	9.46	0.664
Mild	1 (3.8)	2 (4.8)	2 (4.5)	6 (5)	2 (3.4)		
Moderate	1 (3.8)	0 (0)	0 (0)	3 (2.5)	0 (0)		
Severe	1 (3.8)	0 (0)	0 (0)	1 (0.8)	0 (0)		
**Daytime dysfunction**							
None	7 (26.9)	6 (14.3)	4 (9.1)	43 (35.8)	19 (32.8)	33.35	< 0.001**
Mild	7 (26.9)	26 (61.9)	23 (52.3)	50 (41.7)	23 (39.7)		
Moderate	7 (26.9)	9 (21.4)	15 (34.1)	24 (20)	12 (20.7)		
Severe	5 (19.2)	1 (2.4)	2 (4.5)	3 (2.5)	4 (6.9)		
**Total PSQI global score**						3.33	0.504
PSQI > 5, poor sleep quality	21 (80.8)	30 (71.1)	35 (79.5)	95 (79.2)	50 (86.2)		
PSQI < 5, good sleep quality	5 (19.2)	12 (28.6)	9 (20.5)	25 (20.8)	8 (13.8)		

*PSQI*: Pittsburgh Sleep Quality Index, χ^2^: Pearson Chi-square value. *Statistical significance at p < 0.05; **Statistical significance level at p < 0.01; ***Statistical significance at p < 0.001.

The descriptive statistics indicated that the fifth-year students had the highest Global PSQI mean score (M = 8.44, SD = 2.77), followed by year I (M = 8.15, SD = 3.31), year III (M = 8, SD = 3.03), Year IV (M = 7.6, SD = 2.65) and year II (M = 6.64, SD = 2.63). These results indicate that participants in year V and year I presented the worst sleep quality compared to those from other classes. The use of sleeping medication had the lowest mean score (M = 0.09, SD = 039) indicating the least problems while habitual sleep efficiency had the highest mean score, (M = 2.06, SD = 1.38), indicating more problems.

### Comparison of the seven components and the global score of Pittsburgh Sleep Quality Index

The results of one-way ANOVA indicated a significant difference in the scores of sleep quality of medical students belonging to different classes for the seven components of sleep quality and PSQI total score: Subjective sleep quality (F = 3.35, p = 0.011), habitual sleep efficiency (F = 10.20, p < 0.001), daytime dysfunction (F = 3.60, p = 0.007), and PSQI global score (F = 2.76, p = 0.028). However, no significant difference found in the scores for sleep latency, sleep duration, sleep disturbances, and use of sleeping medication (Table 3).

**Table 3:** Comparison of the seven components and the global score of Pittsburgh Sleep Quality Index.

Sleep component	Mean square	F value	p-value
Subjective sleep quality	1.65	3.35	0.011*
Sleep latency	0.12	0.20	0.941
Sleep duration	0.87	2.20	0.070
Habitual sleep efficiency	17.21	10.20	< 0.001***
Sleep disturbances	0.23	0.50	0.733
Sleeping medication	0.25	1.64	0.164
Daytime dysfunction	2.47	3.60	0.007**
PSQI global score	21.27	2.76	0.028*

*p < 0.05; **p < 0.01; ***p < 0.001; *PSQI*: Pittsburgh Sleep Quality Index.

### Significance of the seven components and the global score of Pittsburgh Sleep Quality Index between classes

As in our case, the data exhibit equal variance (as suggested by Lavene’s Statistics), Post Hoc test was selected to determine which classes were significantly different from others. There were significant scores differences in subjective sleep quality between class I and II (p = 0.043) and between class I and IV (p = 0.016); habitual sleep efficiency between class V and all other classes (p < 0.001); daytime dysfunction between class III and IV (p = 0.023) and the PSQI global score between class II and V (p = 0.024) (Table 4).

**Table 4:** p-values of comparison of mean score of the seven components and the global score of Pittsburgh Sleep Quality Index between classes.

Class	Comp I	Comp II	Comp III	Comp IV	Comp V	Comp VI	Comp VII	PSQI
Class I vs Class II	0.043*	0.909	0.740	0.791	0.372	0.613	0.199	0.264
Class I vs Class III	0.918	0.674	0.074	0.905	0.908	0.561	0.831	0.903
Class I vs Class IV	0.016*	0.839	0.078	0.092	0.447	0.212	0.063	0.855
Class I vs Class V	0.907	0.913	0.320	< 0.001***	0.298	0.34	0.609	0.569
Class II vs Class III	0.167	0.726	0.092	0.801	0.368	0.98	0.061	0.395
Class II vs Class IV	0.881	0.686	0.079	< 0.001***	0.745	0.249	0.215	0.889
Class II vs Class V	0.119	0.789	0.296	< 0.001***	0.909	0.888	0.126	0.024*
Class III vs Class IV	0.124	0.401	0.319	< 0.001*	0.442	0.249	0.023*	0.958
Class III vs Class V	0.929	0.516	0.239	< 0.001***	0.277	0.888	0.513	0.41
Class IV vs Class V	0.709	0.909	0.368	0.001***	0.611	0.148	0.343	0.497

*p < 0.05; **p < 0.01; ***p < 0.001; *Comp I* subjective sleep quality, *Comp II* sleep latency, *Comp III* sleep duration, *Comp IV* habitual sleep efficiency, *Comp V* sleep disturbances, *Comp VI* use of sleeping medications, *Comp VII* daytime dysfunction.

## Discussion

This study evaluated the sleep quality of undergraduate medical students in Rwanda using the Pittsburgh Sleep Quality Index (PSQI) and compared the seven components and global score of Pittsburgh Sleep Quality Index across classes. We found a high prevalence of poor sleep quality, 80%, with a significant difference between classes where 86.2% and 80.8% of final and first-year students respectively had poor sleep quality. Our results replicate the findings of studies conducted in Kazakhstan and Brazil which respectively reported that 79.2% and 80.95% of medical students had poor sleep quality^4,30^. However, the current study’s prevalence of poor sleep quality is comparatively higher than what has been reported in similar studies^8,14,22,31^. In Malaysia and Saudi Arabia, 44.23% and 63.2% of medical students reported poor sleep quality respectively^8,22^. In SSA countries like in Ethiopia and Nigeria, 55.8% and 32.5% medical students respectively had poor sleep quality^14,31^. The current study's poor sleep quality may be explained by the stress levels that medical students in Rwanda experience^32^, post-conflict situations^20^ or the COVID-19 pandemic that heightened online learning^33,34^. like our study, research from North India found that first-year students reported having worse sleep quality^35^. The poorest sleep quality reported among final and first-year students may be justified by several clinical rotations that come with financial distress during this period for final-year students, and countless encountered challenges like new schedules, unfamiliar environments, and academic demands for first-year medical students^8,32^. Consistently, scholars revealed that last year’s medical students encounter financial trouble that raises their stress levels^32^, in turn worsening their sleep quality^36^. Thus, actions must be taken among final-year students in Rwanda to address this grave problem since their poor sleep quality may jeopardize the lives of the patients they monitor during their clinical rotations. Similarly, first-year medical students need induction activities to promote their health for sleep hygiene.

Despite the recommendation that sleep duration per night should be 7 h or above for younger adults^5,6^, our study participants slept 5.5 h per night (on average) with 87% sleeping fewer than 7 h. In congruence with our findings, studies conducted in Saudi Arabia, and Slovenia showed that medical students respectively slept 5.8 h and 5.84 h on average^18,37^. Moreover, our results were in line with the findings that 87.6% of medical students slept less than 7 h per night in Pakistan^1^. Worryingly, sleep less than 7 h is associated with poorer general health and increased risk or presence of disease^35^. It has been also studied that sleep deprivation among medical students leads to sleepiness during the daytime and contributes to medical errors, road traffic accidents, and a decrease in academic performance^15^. Regrettably, more than half (53.5%) in the current study had difficulties falling asleep, taking longer than 15 min. Comparatively, a higher proportion of medical students in Saudi Arabia (65.1%) and Brazil (72%), respectively, reported taking more than 15 min to fall asleep^38,39^. The predictors of sleep difficulties among Mexican medical students have been found as symptoms of stress, anger, worry, cognitive hyperarousal, and hypervigilance^40^. Similarly, medical students in Rwanda reported mild to moderate levels of stress^32^. Nevertheless, medical students still sacrifice their sleeping hours to study because of their excessive academic burden^12^. Therefore, open discussions between medical students and academic staff are needed to identify ways to alleviate potential causes that contribute to fewer hours of sleep among medical students in Rwanda.

Mild to moderate sleep disturbances were found among 84.5% of medical students in the current study. Comparatively, this rate is higher than the global prevalence of sleep disturbances (76.8%) in medical students^40^. These results are also higher compared to a study conducted at an Italian University revealing that 63% of medical students had symptoms of sleep disturbances^9^. However, the results from this study are lower than those shown in similar African studies in Ethiopia (95.1%) and Egypt (93.4%)^31,41^. Though the current study reported lower sleep disturbances compared to other African countries, the rate is still worrisome, and thus, it should be kept much lower because of the studied relationship between sleep disturbances, and academic performance^40^. According to some literature, issues of sleep disturbances are the possible markers of current and future psychiatric problems among medical students^40,42^. Further studies also documented that sleep disturbances among medical students not only put them at risk of psychiatric problems but also affect their cognitive skills, emotional intelligence, and academic performance^12^. The current study also revealed that daytime dysfunctions were at 73% with significant differences between classes, notably between class III and IV (p = 0.023), in which third-year students reported more daytime dysfunctions than others, 91%. The results of a significant difference in daytime dysfunctions between different classes agree with a study in Brazil that revealed similar findings^39^. However, the prevalence is higher compared to a study conducted in Jordan that found a prevalence of 50%^3^. Daytime sleep dysfunctions are known to cause medical errors and decrease academic performance^15^. In Rwanda, measures such as regular counseling and education to address daytime sleep dysfunctions among medical students are critical to prevent medical errors as well as improve their behaviors and lifestyle for better academic performance.

Despite a higher prevalence of poor sleep quality in the current study, 79% of medical students classified their subjective sleep quality from fairly to very good. However, their habitual sleep efficiency was found poor whereby 68% of medical students had less than 65% of habitual sleep efficiency. This component was found to be even the most impaired sleep component, which is contrary to an Iranian study which found that habitual sleep efficiency was the best sleep component^11^. Close to our findings, a study in Saudi Arabia reported that 76.1% of medical students classified their subjective sleep quality from fairly good to very good^38^. Also, in India, 74.7% reported their subjective sleep quality from fairly to very good^35^. Contrary to our findings, in Malaysia, 76.1% of medical students had better habitual sleep efficiency which was above 85%^8^. Lack of enough recreational lessons in their annual curriculum and unpredictable school activities as reported in a study in Rwanda might be some of the reasons for this difference^32^. The current study found that both subjective sleep quality and habitual sleep efficiency were significantly different between classes with final-year students significantly presenting the poorest habitual sleep efficiency. These results are in line with previous studies done in India and Brazil which also found these components significantly different among various phases of medical course^35,39^. Moreover, they agree with a prior study conducted in North India which reported that first-year medical students had worse subjective sleep quality compared to other classes^35^. More medical students in the first year 34.6%, significantly experienced a worse subjective sleep quality and this is close to a study in India which found that 35.4% first year medical students reported poor subjective sleep quality^35^. The probable reasons might be that first-year medical student are provided several tasks while they are being reintegrated in a new community which could challenge them in combining several tasks.

Remarkably, the current study found lower use of sleeping medication at 6.6%. When compared to other sleep components, it was even the least impaired. Like our findings, 6.3% and 6% in India and Nepal respectively used sleeping medications^43,44^. Contrarily, higher rates of the usage of sleep medications among medical students were previously reported in Jordan at 21.4%^45^. Similarly, in Saudi Arabia, 24.9% of medical students reported using sleeping medication^46^. In Ethiopia, the use of sleeping medication among medical students was standing at 8.8%^31^. The minimal usage of sleeping medications in the current study is a relief, as sleeping medications have been shown to impair sleep structure and both physical and psychological dependence often follow the use of sleeping medication^47^. That is why even the least usage reported in the current study should be investigated and addressed.

## Strengths and limitations

This study was the first of its kind conducted to the best knowledge of the authors. It was conducted countrywide, and this gives strength to the study as it presents a general picture of the sleep quality among medical students in Rwanda. However, we experienced some limitations: First, though this study used a self-reported scale measuring sleep quality that is psychometric sound as well as fitting well with our context, the participants might give socially desired answers on sensitive questions or not correctly respond some questions as they might not understand^48^. Second, during this study, the education sector was recovering from delays caused by lockdowns of COVID-19, thus medical students had both online and virtual classes that could affect their sleep quality. Third, because this study did not examine the factors that might contribute to poor sleep quality among medical students, more research is needed to examine these factors among medical students in Rwanda.

## Conclusion

In overall, the prevalence of poor sleep quality was alarming in medical students with some participants reporting using medication to fall asleep. Most medical students had less than recommended hours of sleep and their habitual sleep efficiency was the most impaired. However, final-year and first-year students experienced the poorest sleep quality compared to other classes. A large number of medical students suffer from mild to severe daytime dysfunctions. Despite overall poor sleep quality, we found lesser use of medications to fall asleep. Based on these findings, intervention methods such as sleep education, behavioural changes, and relaxation techniques are suggested to address the factors that contribute to poor sleep quality. To address this sleep issue, it is also critical that health promotion policies and strategies, particularly those focusing on healthy sleep hygiene, can be implemented. Though factors linked to modern technologies like the use of social media or more time spent on screens are globally known as the main factors leading to poor sleep quality among medical students^11,49^, future studies should consider psychosocial, and environmental factors that contribute to poor sleep quality among medical students, as well as conduct a prospective study to determine the cause-effect relationship of risk factors for poor sleep quality.

## Data Availability

All relevant data were included in the manuscript. However, data may be shared upon reasonable request and is provided to the corresponding author.

## Abbreviations

AUCA: Adventist University of Central Africa
IRB/CMHS: Institutional Review Board of College of Medicine, and Health Sciences
PSQI: Pittsburgh Sleep Quality Index
UGHE: University of global health equity
UR: University of Rwanda

## References

[1] Arshad AR, Khan MH, Hasan F, Khan SK, Ijaz F, Aftab RK (2021). Quality of sleep and sleep disorders among medical students at a private medical college in Lahore, Pakistan. Adv. Biores..

[2] Paudel K, Adhikari TB, Khanal P, Bhatta R, Paudel R, Bhusal S (2022). Sleep quality and its correlates among undergraduate medical students in Nepal: A cross-sectional study. PLoS Glob. Public Health.

[3] Alqudah M, Balousha SAM, Balusha AAK, Al-U’dat DG, Saadeh R, Alrabadi N (2022). Daytime sleepiness among medical colleges’ students in Jordan: Impact on academic performance. Sleep Disord..

[4] Saygın M, Öztürk Ö, Gonca T, Has M, Hayri UB, Kurt Y (2016). Investigation of sleep quality and sleep disorders in students of medicine. Turk Toraks Dergisi.

[5] Watson NF, Badr MS, Belenky G, Bliwise DL, Buxton OM, Buysse D (2015). Recommended amount of sleep for a healthy adult: A joint consensus statement of the American Academy of Sleep Medicine and Sleep Research Society. Sleep.

[6] Hirshkowitz M, Whiton K, Albert SM, Alessi C, Bruni O, DonCarlos L (2015). National Sleep Foundation’s sleep time duration recommendations: Methodology and results summary. Sleep Health.

[7] Alghannami A, Alrashed A, Alshehri R, Alotaibi S, Alharbi M, Mukaddem A (2021). The sleep pattern of medical students: Examining the impact of excessive Internet use. Int. J. Med. Dev. Ctries..

[8] Farah Natashah Mohd, A., Manh, A. & Hanapiah, M. H. Poor sleep quality among medical students in International Islamic University Malaysia (IIUM) and its association with mental health and other factors. *IMJM* **19** (2020).

[9] Belingheri M, Pellegrini A, Facchetti R, de Vito G, Cesana G, Riva MA (2020). Self-reported prevalence of sleep disorders among medical and nursing students. Occup. Med. (Chic., Ill.).

[10] Ibrahim NK (2017). Sleep quality among medical students at King Abdulaziz University: A cross-sectional study. J. Community Med. Health Educ..

[11] Mohammadbeigi, A., Absari, R., Valizadeh, F., Saadati, M., Sharifimoghadam, S., Ahmadi, A. *et al*. Sleep quality in medical students; the impact of over-use of mobile cell-phone and social networks. Article Information Abstract [Internet]. *JRHS J. Res. Health Sci.* **16** (2016). http://www.umsha.ac.ir/jrhs. PMC7189085 27061997

[12] Javaid R, UlMomina A, Sarwar MZ, Naqi SA (2020). Quality of sleep and academic performance among medical university students. J. Coll. Physicians Surg. Pak..

[13] Wondie T, Molla A, Mulat H, Damene W, Bekele M, Madoro D (2021). Magnitude and correlates of sleep quality among undergraduate medical students in Ethiopia: Cross-sectional study. Sleep Sci. Pract..

[14] James B, Omoaregba J, Igberase O (2011). Prevalence and correlates of poor sleep quality among medical students at a Nigerian university. Ann. Niger. Med..

[15] Alhazzani N, Masudi E, Algarni A (2018). The relationship between sleep patterns and academic performance among medical students at King Saud Bin Abdulaziz University for Health Sciences. Egypt. J. Hosp. Med..

[16] Siraj HH, Salam A, Roslan R, Hasan NA, Jin TH, Othman MN (2014). Sleep pattern and academic performance of undergraduate medical students at Universiti Kebangsaan Malaysia. J. Appl. Pharm. Sci..

[17] Abdulghani HM, Alrowais NA, Bin-Saad NS, Al-Subaie NM, Haji AMA, Alhaqwi AI (2012). Sleep disorder among medical students: Relationship to their academic performance. Med. Teach..

[18] Almojali AI, Almalki SA, Alothman AS, Masuadi EM, Alaqeel MK (2017). The prevalence and association of stress with sleep quality among medical students. J. Epidemiol. Glob. Health [Internet].

[19] Isobel S, McCloughen A, Goodyear M, Foster K (2021). Intergenerational trauma and its relationship to mental health care: A qualitative inquiry. Community Ment. Health J. [Internet].

[20] Rudahindwa S, Mutesa L, Rutembesa E, Mutabaruka J, Qu A, Wildman DE (2018). Transgenerational effects of the genocide against the Tutsi in Rwanda: A post-traumatic stress disorder symptom domain analysis. AAS Open Res..

[21] Lopez, A. D., Mathers, C. D., Ezzati, M., Jamison, D. T. & Murray, C. J. Global and regional burden of disease and risk factors, 2001: Systematic analysis of population health data. *Lancet* [*Internet*] **367**(9524), 1747–1757 (2006). http://www.thelancet.com/article/S0140673606687709/fulltext. 10.1016/S0140-6736(06)68770-9 16731270

[22] Al-Khani AM, Sarhandi MI, Zaghloul MS, Ewid M, Saquib N (2019). A cross-sectional survey on sleep quality, mental health, and academic performance among medical students in Saudi Arabia. BMC Res. Notes [Internet].

[23] Vedaa Ø, Erevik EK, Hysing M, Hayley AC, Sivertsen B (2019). Insomnia, sleep duration and academic performance: A national survey of Norwegian college and university students. Sleep Med. X.

[24] Alfadeel MA, Alfadeel MA, Alqahtani N, Alhudaib M, Almudhee S, Aljarbou R (2019). The prevalence of insomnia among female medical students of Almaarefa Colleges in Riyadh City-Kingdom of Saudi Arabia 2015–2016. Indo Am. J. Pharm. Sci..

[25] Naing NN (2003). Determination of sample size. Malays. J. Med. Sci..

[26] Buysse, D. J., Reynolds, C. F., Monk, T. H., Berman, S. R. & Kupfer, D. J. PSQI.pdf [Internet]. Oakland Psychiatric Associate. http://www.opapc.com/uploads/documents/PSQI.pdf.

[27] Al-Khani AM, Sarhandi MI, Zaghloul MS, Ewid M, Saquib N (2019). A cross-sectional survey on sleep quality, mental health, and academic performance among medical students in Saudi Arabia. BMC Res. Notes.

[28] Armstrong RA (2014). When to use the Bonferroni correction. Ophthalmic Physiol. Opt..

[29] Shrestha, B. & Dunn, L. The declaration of Helsinki on medical research involving human subjects: A review of seventh revision. *J. Nepal Health Res. Counc.* [*Internet*] **17**(4), 548–552 (2020). https://pubmed.ncbi.nlm.nih.gov/32001865/. 10.33314/jnhrc.v17i4.1042 32001865

[30] de Almeida FVQ, Tarlane B, Silva S, das Graças B, Paiva O, Montina CB (2020). Influence of sleep quality on academic performance of medical students. Rev. Soc. Bras. Clin. Med..

[31] Lemma S, Gelaye B, Berhane Y, Worku A, Williams MA (2012). Sleep quality and its psychological correlates among university students in Ethiopia: A cross-sectional study. BMC Psychiatry.

[32] Kubwimana L, Mutatsineza G, Tesi L, Wong R (2022). Assessing the stress level among medical students in Rwanda. Open J. Psychiatry.

[33] Marelli S, Castelnuovo A, Somma A, Castronovo V, Mombelli S, Bottoni D (2021). Impact of COVID-19 lockdown on sleep quality in university students and administration staff. J. Neurol..

[34] Saguem BN, Nakhli J, Romdhane I, Nasr SB (2022). Predictors of sleep quality in medical students during COVID-19 confinement. Encephale.

[35] Gupta S, Bhardwaj A, Nadda A, Gill A, Mittal A, Gupta S (2020). A comparative study of sleep quality in different phases of the medical course: A study from Haryana (North India). J. Fam. Med. Prim. Care.

[36] Safhi M, Alafif R, Alamoudi N, Alamoudi M, Alghamdi W, Albishri S (2020). The association of stress with sleep quality among medical students at King Abdulaziz University. J. Fam. Med. Prim. Care.

[37] Ulen, K., Dž, A., Aferovic, A., Ulen, K. & Mangaloiu, D. Sleep habits among medical students and correlation between sleep quality and academic performance smoking prevalence and risk perception among Romanian medical students: Tobacco versus E-cigarette [Internet]. https://academic.oup.com/eurpub/article/28/suppl_4/cky214.141/5185766.

[38] Siddiqui AF, Al-Musa H, Al-Amri H, Al-Qahtani A, Al-Shahrani M, Al-Qahtani M (2016). Sleep patterns and predictors of poor sleep quality among medical students in King Khalid university, Saudi Arabia. Malays. J. Med. Sci..

[39] de Corrêa C, de Oliveira FK, Pizzamiglio DS, Ortolan EVP, Weber SAT (2017). Qualidade de sono em estudantes de medicina: Comparação das diferentes fases do curso. J. Bras. Pneumol..

[40] Azad MC, Fraser K, Rumana N, Abdullah AF, Shahana N, Hanly PJ (2015). Sleep disturbances among medical students: A global perspective. J. Clin. Sleep Med..

[41] Elwasify M, Barakat DH, Fawzy M, Elwasify M, Rashed I, Radwan DN (2016). Quality of sleep in a sample of Egyptian medical students. Middle East Curr. Psychiatry.

[42] Dugas EN, Sylvestre MP, O’Loughlin EK, Brunet J, Kakinami L, Constantin E (2017). Nicotine dependence and sleep quality in young adults. Addict. Behav..

[43] Shrestha D, Rajbhandari P (2018). Sleep Quality among medical students of a tertiary care hospital: A descriptive cross-sectional study. J. Nepal Med. Assoc..

[44] Dalui SK, Datta A, Ghosh A, Biswas S, Roy UK, Biswas S (2017). Self-medication of sleeping pills among MBBS students in a medical college of West Bengal, India. Int. J. Basic Clin. Pharmacol..

[45] Alqudah M, Balousha SAM, Al-Shboul O, Al-Dwairi A, Alfaqih MA, Alzoubi KH (2019). Insomnia among medical and paramedical students in Jordan: Impact on academic performance. Biomed Res. Int..

[46] Alasmari MM, Alkanani RS, Alshareef AS, Alsulmi SS, Althegfi RI, Bokhari TA (2022). Medical students’ attitudes toward sleeping pill usage: A cross-sectional study. Front. Psychiatry [Internet]..

[47] Coates, T. J. & Thoresen, C. E. What to use instead of sleeping pills. *JAMA* [*Internet*] **240**(21), 2311–2312 (1978). https://jamanetwork.com/journals/jama/fullarticle/362384. 702764

[48] Demetriou C, Ozer BU, Essau CA (2015). Self-report questionnaires. Encycl. Clin. Psychol. [Internet]..

[49] Tahir MJ, Malik NI, Ullah I, Khan HR, Perveen S, Ramalho R (2021). Internet addiction and sleep quality among medical students during the COVID-19 pandemic: A multinational cross-sectional survey. PLoS One.

